# The prebiotic potential of dietary onion extracts: shaping gut microbial structures and promoting beneficial metabolites

**DOI:** 10.1128/msystems.01189-24

**Published:** 2024-12-23

**Authors:** Yebeen Yoo, Seongok Kim, WonJune Lee, Jinwoo Kim, Bokyung Son, Kwang Jun Lee, Hakdong Shin

**Affiliations:** 1 Department of Food Science and Biotechnology, College of Life Science, Sejong University, Seoul, South Korea; 2 Carbohydrate Bioproduct Research Center, Sejong University, Seoul, South Korea; 3 Department of Food Biotechnology, Dong-A University, Busan, Republic of Korea; 4 Division of Zoonotic and Vector Borne Diseases Research, Center for Infectious Diseases Research, National Institute of Health, Cheongju, South Korea; Ilisimatusarfik, Nuuk, Greenland

**Keywords:** onion extracts, gut microbiome, enterotype, short-chain fatty acids, prebiotics, metabolites, tryptophan metabolism

## Abstract

Onions are well-known vegetables that offer various health benefits. This study explores the impact of onion extracts on gut microbiome using an *in vitro* fecal incubation model and metabolome analysis. Fecal samples were collected from 19 healthy donors and incubated in the presence or absence of onion extracts for 24 h. To reduce inter-individual variability in the gut microbiome, we employed enterotyping based on baseline fecal microbiota: 14 subjects with a *Bacteroides*-dominant type (enterotype B) and 5 subjects with *Prevotella*-dominant type (enterotype P). Alpha diversity was significantly reduced in the onion-treated group compared to the non-treated control group in both *Bacteroides*- and *Prevotella*-dominant types. However, significant structural differences in bacterial communities were observed based on weighted UniFrac distance. Notably, short-chain fatty acid (SCFA)-producing bacteria, such as *Bifidobacterium*_388775, *Feacalibacterium*, and *Fusicatenibacter*, were overrepresented in response to onion extracts in enterotype B. Furthermore, genes related to butyrate production were significantly overrepresented in the onion-treated group within enterotype B. Consistent with the enriched taxa and the predicted metabolic pathways, SCFAs and their related metabolites were significantly enriched in the onion-treated group. Additionally, tryptophan metabolism-derived metabolites, including indolelactate (ILA) and indolepropionate (IPA), were elevated by 4- and 32-fold, respectively, in the onion-treated group compared to the control group. *In vitro* growth assays showed an increase in lactobacilli strains in the presence of onion extracts. These results provide evidence that onion extracts could serve as promising prebiotics by altering gut microbial structure and promoting the production of beneficiary metabolites, including SCFAs and indole derivatives, and enhancing the growth of probiotics.

**IMPORTANCE:**

This study is significant as it provides compelling evidence that onion extracts have the potential to serve as effective prebiotics. Utilizing an *in vitro* fecal incubation model and enterotyping to reduce inter-individual variability, the research demonstrates how onion extracts can alter gut microbial structure and promote the production of beneficial metabolites, including SCFAs and indole derivatives like ILA and IPA. Additionally, onion extract treatment enhances the growth of beneficial probiotics. The findings underscore the potential of onion extracts to improve gut health by enriching specific beneficial bacteria and metabolic pathways, thereby supporting the development of functional foods aimed at improving gut microbiota composition and metabolic health.

## INTRODUCTION

*Allium cepa*, commonly known as onion, is a bulbous plant that is extensively cultivated worldwide. Compounds from onion have been reported to offer a range of health benefits, including anti-carcinogenic, anti-platelet activity, anti-thrombotic activity, anti-asthmatic, and antibiotic effects (1). These compounds are phytomolecules, including various carbohydrates, polyphenols, dietary fibers, flavonoids, and sulfur compounds (2–5). Among them, carborhydrates such as oligosaccharides have gained significant attention for their prebiotic application, as its supplementation has been shown to improve human health disorders, including Chron’s disease, allergen reaction, atopic dermatitis, and intestinal diseases (6–11).

Prebiotics have recently been defined by the International Scientific Association for Probiotics and Prebiotics as “a substrate that is selectively utilized by host microorganisms conferring a health benefit (12).” Substrates considered prebiotics include non-digestible oligosaccharides, such as fructooligosaccharide (FOS) and galactooligosaccharide, dietary fiber like inulin and pectin, plant-derived polyphenols, and polysaturated fatty acids (12, 13). These compounds naturally exist in different dietary food products, including asparagus, sugar beet, garlic, chicory, onion, and other vegetables (14). Prebiotics are metabolized by gut microbiota, resulting in the production of beneficial products through microbial cross-feeding, such as short-chain fatty acid and tryptophan-derived metabolites (indolelactate [ILA] and indolepropionate [IPA]). These metabolites have been shown to impact host biological functions, including the maintenance of epithelial barrier integrity, immune modulation, the control of intestinal inflammation, and metabolic disorders (15–23).

The gut microbiota, consisting of 10–100 trillion microorganisms, is considered as an important factor due to its contribution to human health (24). The structure of gut microbiota varies significantly among individuals and is influenced by host factors such as age, nationality, gender, and body mass index (BMI) (25). These variations have posed challenges in studying the relationships between the human microbiome and health. To address this, recent studies have introduced enterotyping, which categorizes complex human gut microbial communities into three distinct enterotypes based on the predominance of specific genera: *Bacteroides* (enterotype 1), *Prevotella* (enterotype 2), and *Ruminococcus* (enterotype 3), although the classification of enterotype 3 remains controversial (25, 26). These enterotypes represent densely populated areas of community composition within a multi-dimensional space, simplifying the complex gut microbial community by minimizing individual variation (25). This approach has facilitated deeper exploration of the connections between the human microbiome and various diseases or diets, which has contributed to advances in cancer diagnostics, implications for weight loss, and the development of personalized nutrition plans for obesity management (27–30).

In this study, we demonstrate the potential of dietary onion extract as a prebiotic, highlighting its significant impact on gut microbial composition and its ability to enhance the production of beneficial metabolites such as butyrate and IPA/ILA.

## MATERIALS AND METHODS

### Fecal sample collection

A total of 19 healthy individuals, ranging from 22 to 34 years of age and who had not taken any antibiotics for at least a month, participated in the study and were instructed to self-collect fecal samples. Sterile cotton-tipped swabs were used for the collection of fresh fecal samples, which were subjected to immediate incubation in an anaerobic chamber for *in vitro* fecal culturing. The metadata, including gender, age, and BMI, were recorded through questionnaires (Table 1).

**TABLE 1:** Characteristics of the participants

	Male	Female	

Number	11	8	
Age (AVG^*a*^ ± SD)	30.3 ± 4.7	24.5 ± 2.5	
BMI (AVG ± SD)	26.3 ± 9.2	21.1 ± 3.0	
Underweight	0	2	
Normal	7	5	
Overweight	3	1	
Obese	1	0	

^*a*^ AVG, average.

### *In vitro* fecal incubation

Onion powder (trade name: Onion powder without additives; manufactured by Clear Fields, Hongcheon County, Gangwon Province, South Korea) was obtained from local market (Seoul, South Korea). It was dissolved in sterile tertiary distilled water at 10% (wt/vol). The basal medium was prepared under anaerobic condition as described previously (31) to mimic human intestine environment. The fecal sample was homogenized in basal medium and filtered through a sterile nylon mesh (1 mm) to make 2% (wt/vol) fecal suspension. This procedure was conducted in an anaerobic chamber (vinyl anaerobic chamber type A, Coy Laboratory Products) where anaerobic conditions were ensured using Oxoid Resazurin Anaerobic Indicator (cat. no. BR0055B; Thermo Fisher Scientific, Waltham, MA, USA). The fecal suspension was inoculated with the prepared onion solution at a final concentration of 1% (wt/vol) and incubated at 37℃ with agitation for 24 h. The fecal suspension without onion extract was used as control. Aliquots from onion-treated or non-treated samples were harvested at 0 and 24 h, respectively, followed by storage at −80℃ until further analysis.

### DNA extraction and 16S rRNA gene sequencing

Total genomic DNA was extracted using DNeasy PowerSoil HTP 96 Kit (QIAGEN, Hilden, Germany). The V4 region of 16S rRNA gene was PCR-amplified using 515F-806R primers (32). The amplicon concentrations were determined using the Quant-iT PicoGreen ds DNA assay kit (Invitrogen, California, USA), and fluorescence was measured using a microplate reader (BioTek, Vermont, USA). Each amplicon was pooled in equimolar proportions into one tube, and the pool was purified using a NucleoSpin PCR clean-up kit (MACHEREY-NAGEL, Düren, Germany). The final pooled amplicons were sequenced using the Illumina MiSeq platform (2  ×  300 cycles, paired end).

### Gut microbiota analysis

The raw sequencing data obtained in FASTQ format were processed using Quantitative Insights into Microbial Ecology 2 (version 2023.05) (33). The reads were quality filtered via the DADA2 pipeline (34) to generate amplicon sequence variant (ASV) tables. The taxonomy of each ASV was assigned using the feature classifier, the naive Bayesian classifier, with the Greengenes2 database. Alpha diversity (Faith’s phylogenetic diversity [PD] index) and beta diversity (unweighted and weighted UniFrac distance) were calculated using q2-diversity. The Kruskal-Wallis test for alpha diversity and permutational multivariate analysis of variance for beta diversity were applied to the statistical analysis. Linear discriminant analysis (LDA score >3.0) was conducted to identify significantly differential taxonomy compared to control. The Phylogenetic Investigation of Communities by Reconstruction of Unobserved States 2 (PICRUSt2, version 2.5.2) (35) was used to predict the functional profiles of the gut microbiome based on the Kyoto Encyclopedia of Genes and Genomes (KEGG) database. In all the analyses, *P* values less than 0.05 were considered statistically significant.

Gut enterotypes were classified based on the relative abundance of taxonomy at the genus level using the Jensen-Shannon divergence (JSD) distance and the partitioning around medoids (PAM) clustering algorithm in the R environment (25). The optimal number of clusters was evaluated using the Calinski-Harabasz (CH) index (36), and the statistical significance was assessed using silhouette coefficients (37).

### Sample preparation for untargeted metabolomic profiles

The fecal sample (500 µL) was extracted by mixing an equal volume of 10% of acetonitrile, followed by vigorous shaking for 1 min, then incubated at −20°C for 2 h. The resulting mixture was centrifuged at 4°C and 13,000 × *g* for 15 min, followed by homogenization using a tissue lyser (QIAGEN) at 30 Hz for 10 min. The supernatant was subjected to liquid chromatography-tandem mass spectrometry (LC-MS/MS) to profile the metabolites, with quality control (QC) samples used in parallel.

### Ultra-high-performance liquid chromatography-tandem mass spectrometry analysis for metabolites profiles

The metabolites derived from fecal microbiota were analyzed using a ultra-high-performance liquid chromatography (U-HPLC) system (Vanquish U-HPLC, Thermo Fisher Scientific), coupled to a quadrupole mass spectrometer (Thermo Scientific Orbitrap Exploris 120 high-resolution/accurate mass spectrometer, interfaced with a heated electrospray ionization source), at the Biopolymer Research Center for Advanced Materials (Sejong University, Seoul, Republic of Korea). The procedures are performed as described previously (38). For the chromatographic separation, the prepared samples (10 µL) were injected into a C18 column (ACQUITY UPLC BEH C18, 2.1 × 100 mm, 1.7 µm; Waters Corp., Milford, MA, USA), with the column temperature maintained at 45°C throughout the acquisition period. The mobile phase was eluted by a gradient of water (A) and acetonitrile (B), containing 0.1% acetic acid, with a gradient dilution profile of 95% A (0–2 min), 95%–5% A (2–15 min), 5% A (15–17 min), 5%–95% A (17–18 min), and 95% A (18–20 min), at a flow rate of 0.3 mL/min. The mass spectrum conditions included a heated capillary of 320°C, a vaporizer temperature of 350°C, a spray voltage of 3.5 kV in positive mode and 2.5 kV in negative mode. The sheath gas, aux gas, and sweep gas were set at 50, 25, and 1 psi, respectively. The higher-energy collisional dissociation collision energy was set at 15%, 30%, and 60%, and the scan range covered 55–700 *m*/*z* in full scan, with a resolution of 120,000 for MS1 and 15,000 for MS2.

The raw mass spectrometry (MS) data were processed using Xcalibur (version 4.6) and Compound Discoverer (version 3.3) (Thermo Fisher Scientific). Normalization of unknown compound intensities using the QC sample was performed. Enrichment analysis to identify the potential KEGG pathway was performed using MetaboAnalyst (https://www.microbiomeanalyst.ca/, accessed on 29 December 2023). Statistical significance was assessed using unpaired *t*-test after a two-stage step-up correction method (39), with *P* values of <0.05 considered statistically significant.

### Analysis of onion powder composition

Onion powder was dissolved in sterile tertiary distilled water at 10% (wt/vol) by voltexing. One milliliter of the resulting solution was transferred into an Eppendorf tube and centrifuged at 4°C and 13,000 × *g* for 15 min. The supernatant was analyzed by ultra-high-performance liquid chromatography-tandem mass spectrometry (U-HPLC MS/MS) to profile its composition. For U-HPLC MS/MS analysis, the procedures followed were the same as those described in U-HPLC MS/MS analysis for metabolite profiles.

### The analysis of co-occurrence networks

The Cytoscape plug-in CoNet (version 1.1.1 beta) was used for network construction, and networks were conducted as previously described (40). Briefly, networks were developed using an ensemble approach that integrates various correlation measures, including Spearman’s rank correlation coefficient, mutual information similarities, and distance metrics (Bray-Curtis and Kullback-Leibler). The co-occurrence patterns were then visualized as networks in Cytoscape (version 3.9.1) using an organic layout. Statistical analysis was conducted with the Network Analyzer tool, thresholding Spearman’s rank correlation coefficient of >0.4 and a Benjamini-Hochberg corrected *P* value (false discovery rate < 0.05) for significance.

### *In vitro* growth of probiotics

The bacterial strains used in this study are listed subsequently (see Table 3). All lactobacilli strains were cultured in De Man, Rogosa, and Sharpe (MRS) broth (BD Difco, Maryland, USA). The broth, with or without onion powder at a final concentration of 1% (wt/vol), was prepared by autoclaving according to manufacturer’s instructions and cooled down and supplemented with L-cysteine at a final concentration of 0.05% (wt/vol). For culturing lactobacilli strains, a 100-µL aliquot of each strain was inoculated in 10 mL of the prepared MRS broth and incubated at 37°C for 4 days under anaerobic condition for preactivation. The MRS media, with or without onion extracts, were aliquoted into a 96-well microplate (198 µL per well), and the preactivated probiotics, adjusted to an OD600 of 1.5, were added at a final concentration of 1% (vol/vol). The plates were incubated at 37°C for 24 h under anaerobic condition. The absorbance at 600 nm was measured at the indicated time points.

## RESULTS

### Characteristics of the study participants

Nineteen adults, aged 22–34, with a BMI ranging from 18.42 to 27.47 kg/m^2^, were enrolled in this study. All subjects had not been prescribed antibiotics for 1 month prior to the experiment. The general characteristics of all participants are listed in Table 1.

### Identification of enterotypes among all participants

The total of sequence reads was obtained from fecal samples, with an average of 16,834 ± 11,899 sequence reads per sample. These reads were binned into ASVs (Table 2).

**TABLE 2:** Sequencing information for each group

Group	Control		Onion	Total
	0 h	24 h		
No. of samples	19	19	19	57
No. of sequences	358,445	461,279	139,835	959,559
Average sequence ± SD	18,866 ± 11,169	24,278 ± 12,483	7,360 ± 1,524	16,834 ± 11,899
No. of unique features (ASVs)	501	612	252	727
Average features (ASVs) ± SD	85 ± 25	109 ± 31	45 ± 11	80 ± 36

**Fig 1:**
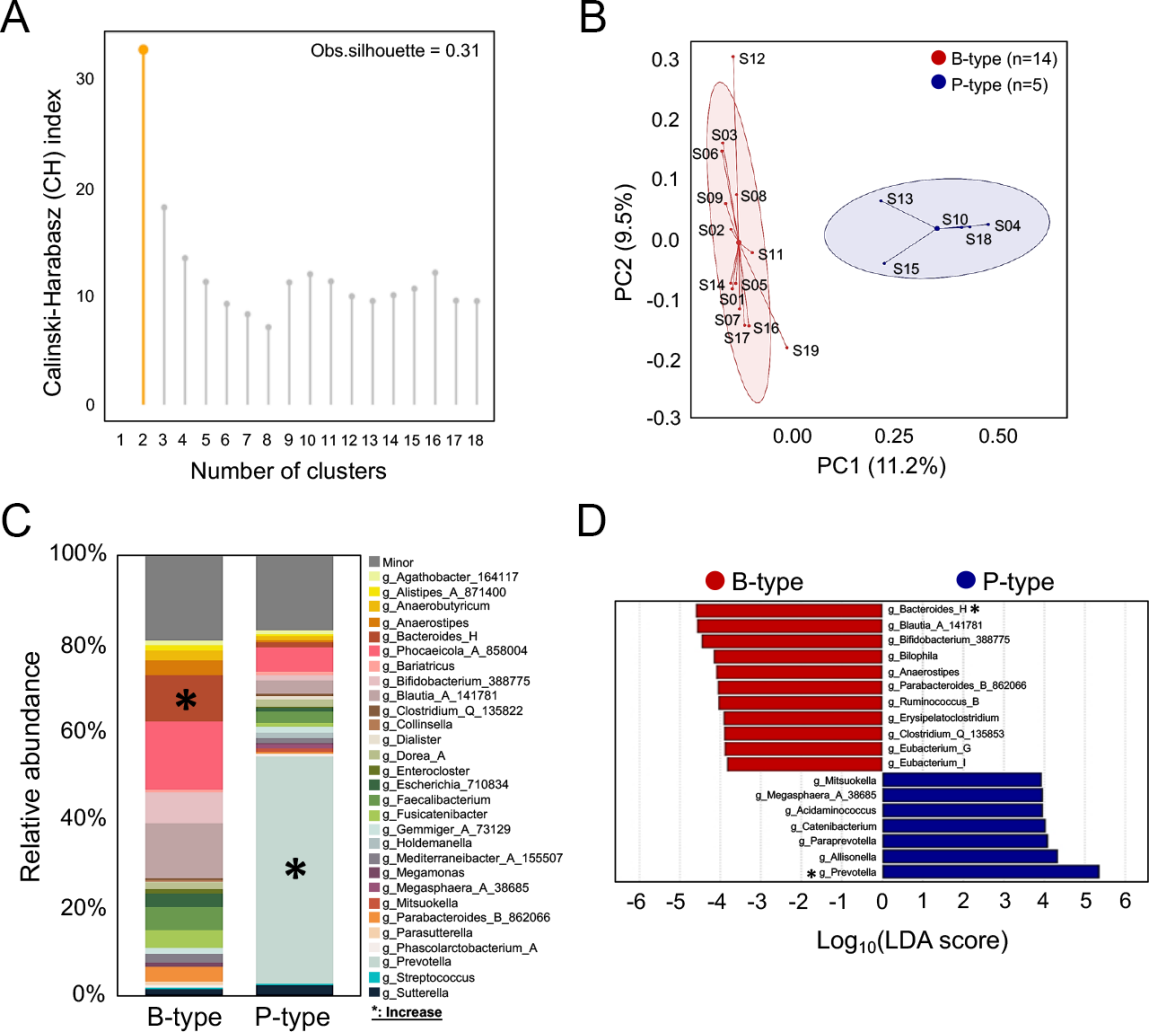
Gut enterotyping of all participants. (**A**) Calinski-Harabasz (CH) index to determine the optimal number of clusters. (**B**) Principal coordinate analysis plot showing enterotype of subjects based on relative genus abundance using the Jensen-Shannon divergence distance and partitioning around medoids clustering algorithm. Samples were color-coded by enterotyping results. Covariance ellipses were projected for each cluster, and bound of cluster was marked two standard deviations (2σ) in each direction from the mean of cluster. Red represents *Bacteroides*-dominant enterotype and blue represents *Prevotella*-dominant enterotype, respectively. (**C**) Bacterial taxa plot based on relative abundance in each enterotype. The black asterisks indicate the most statistically abundant taxa in either B- or P-type. (**D**) Histogram showing differentially abundant taxa between enterotypes using LefSe (LDA scores > 3 and adjusted *P* value of <0.05). Negative (red bars) and positive (blue bars) LDA scores indicate overrepresented genus in either enterotype B or P, respectively.

To deconvolve inter-individual variability across all participants for a clearer understanding of the impact of onion extracts on gut microbial structures, we determined the enterotype of each subject using control samples. Specifically, enterotyping is a well-established classification approach for evaluating dietary effects on the gut microbiome and controlling inter-individual variability (40, 41). By clustering individuals with similar microbiome compositions, enterotyping reduces variability and allows for more precise analysis of diet-microbiome interactions. Clustering was performed based on the relative abundance at genus level using JSD distance and the PAM clustering algorithm. The optimal number of clusters was estimated via the CH index, as shown in Fig. 1A. The structure of microbiota from all samples was separated into two distinct clusters (Fig. 1B, 14 subjects with a *Bacteroides*-dominant type and 5 subjects with *Prevotella*-dominant type). To further corroborate the enterotype classification across all subjects, linear discriminant analysis effect size (LefSe) analysis was employed to find the principal taxonomic drivers for each enterotype. In the *Bacteroides*-dominant group, the *Bacteroides* genus is notably abundant, mirroring the pattern in the *Prevotella*-dominant group (Fig. 1C and D). Furthermore, the identified driver taxa were significantly represented in the microbiota of subjects within each enterotype group (Fig. S1).

### The effect of onion extracts on microbial diversity and structure differences

**Fig 2:**
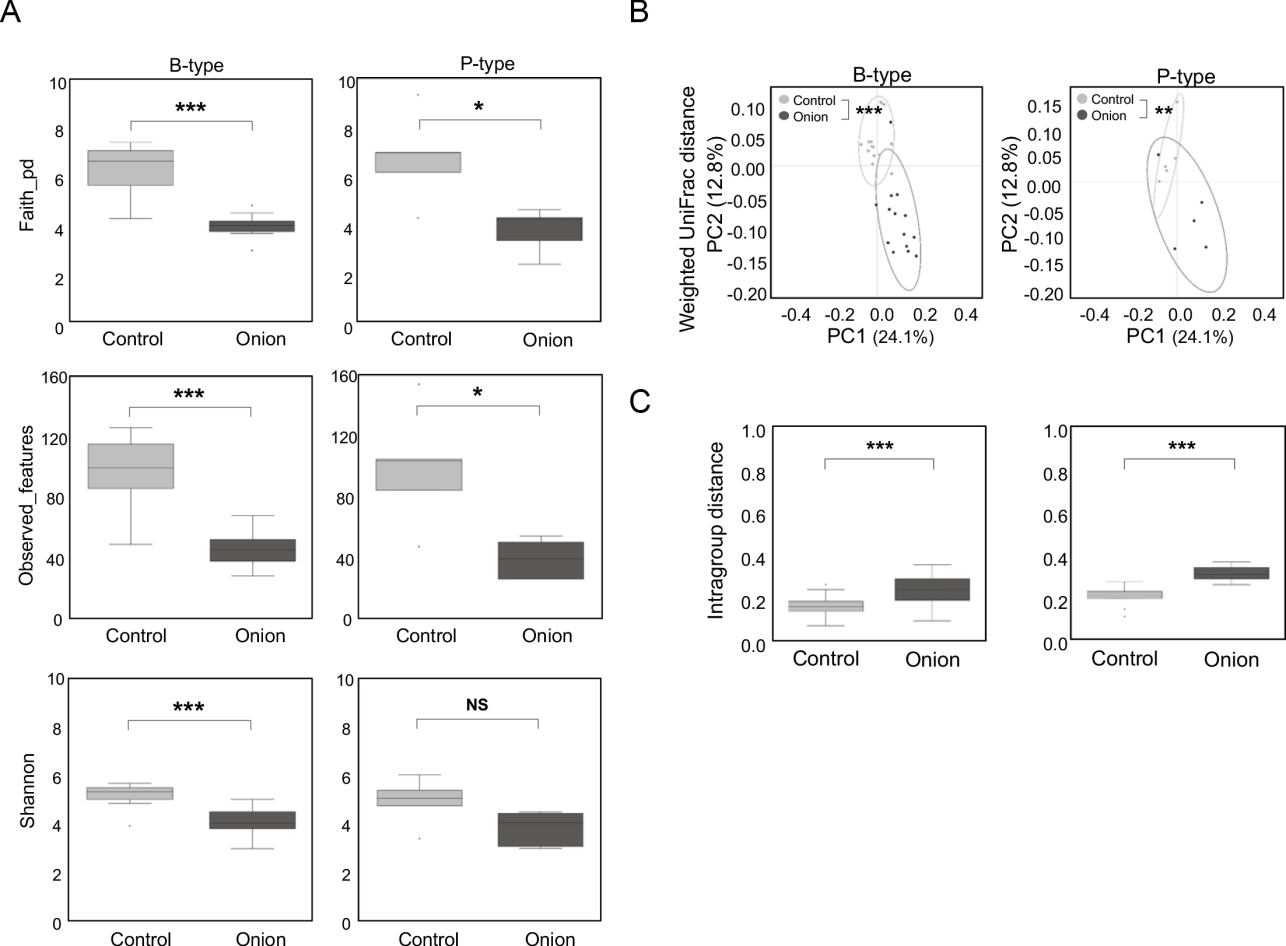
The effect of onion extracts on changes in gut microbial structure according to enterotype. (**A**) Alpha diversity represented by Faith’s PD index, observed features, and Shannon’s metrics. Data are shown in median ± 25th percentile, Mann-Whitney test. NS, nonsignificant, *P < 0.05,, ***P < 0.001. (**B**) Beta diversity evaluated by weighted UniFrac distance. Permutational multivariate analysis of variance was used to test statistical differences between control and onion-treated groups, Mann-Whitney test. **P < 0.01, ***P < 0.001. (**C**) Intragroup distance using weighted UniFrac distance between groups. Data shown and error bars are mean ± SEM (non-parametric *t*-test). ***P < 0.001.

To assess the impact of onion extract on gut microbial diversity, alpha diversity was evaluated using Shannon’s index, observed feature counts, and Faith’s PD index. Across all alpha diversity metrics, the onion-treated group showed a significant reduction in microbial richness and evenness compared to that of non-treated control group in both *Bacteroides*- and *Prevotella*-dominant types, respectively (Fig. 2A).

To investigate whether onion extracts could induce the changes in bacterial communities, principal coordinate analysis (PCoA) analysis was performed based on weighted UniFrac distance. Each group showed well separation and the community structure in the onion-treated group significantly differed from those of control group in both *Bacteroides*- and *Prevotella*-dominant types, respectively (Fig. 2B; permutational multivariate analysis of variance, *P* < 0.001). We then examined intragroup distance between the control and onion-treated groups, revealing that the onion-treated group exhibited statistically significantly greater distance compared to the control group in both *Bacteroides*- and *Prevotella*-dominant types, respectively (Fig. 2C). This strongly suggests an alteration in gut microbial structures in response to onion treatment. Consistent with weighted UniFrac distance, a similar separation and intragroup distances were also observed using unweighted UniFrac distance in the PCoA plot (Fig. S2).

**Fig 3:**
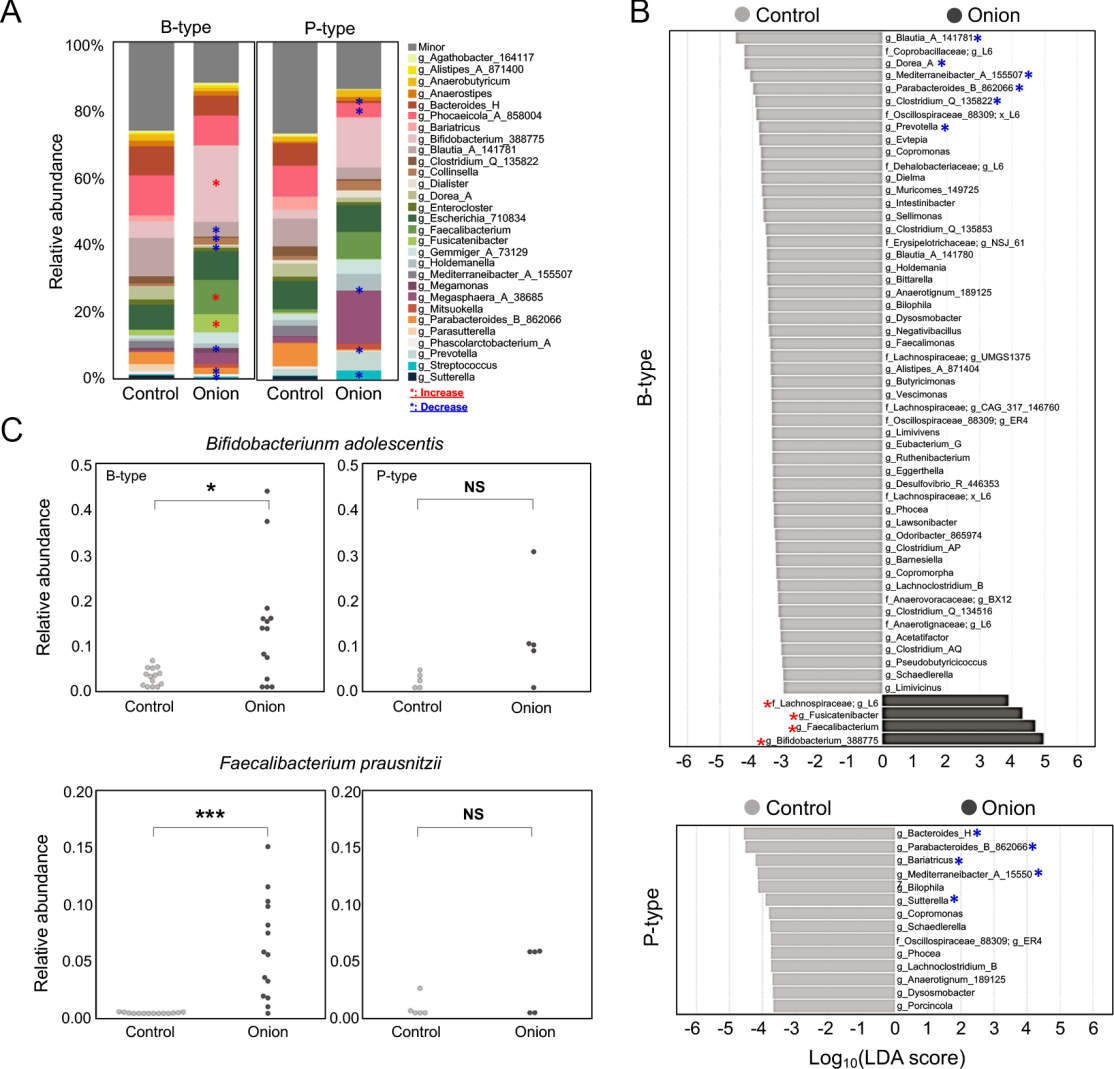
Differences in microbial structure between control and onion groups according to enterotype. (**A**) Microbial taxa plot at genus level between groups in each enterotype. Red and blue asterisks denote differentially enriched or decreased taxa, respectively, as determined by LEfSe (LDA score > 3.0). (**B**) Bar chart showing differentially abundant taxa between groups using LEfSe (LDA score > 3.0 and Benjamini-Hochberg corrected *P* value of Wilcoxon test < 0.05). Red asterisks indicate taxa enriched in the onion-treated group, whereas blue asterisks denote taxa decreased in the onion-treated group compared to the control group. (**C**) Relative abundance of *Bifidobacterium adolescentis* and *Faecalibacterium prausnitzii* in control and onion groups using feature analysis according to enterotype. Statistical analysis was performed using Mann-Whitney test with significance represented as *P < 0.05, **P < 0.01, ***P < 0.001 and non-significant (NS).

We observed changes in microbial composition of the gut microbiota induced by the treatment of onion extract treatment. To further identify which bacterial taxa are differentially increased or decreased in the onion-treated group compared to the control group, we analyzed the relative bacterial abundance at the genus level using LEfSe. Significantly increased bacterial genera in enterotype B following onion extract treatment were *Bifidobacterium*_388775, *Feacalibacterium*, *Fusicatenibacter*, and *Lachnospiraceae*, respectively, while nothing was observed in enterotye *P* (Fig. 3A and B; LDA score > 3.0). In contrast, the bacterial genera that decreased in enterotype B following onion extract treatment were *Blautia*_A_141781, *Coprobacillaceae*, *Dorea_*A, *Mediterraneibacter*_A_155507, *Parabacteroides*_B_862066, *Clostridium*_Q_135822, *Oscillospiraceae*_88309, and *Prevotella*, respectively. In enterotype P, the decreased genera included *Bacteroides*_H, *Parabacteroides*_B_862066, *Bariatricus*, *Mediterraneibacter*_A_155507, and *Sutterella*, respectively (Fig. 3A and B). Given that these taxa were notably enriched taxa in response to onion extract treatment, we further aimed to identify these two genera at strain level. Notably, *Bifidobacterium adolescentis* and *Feacalibacterium prausnitzii*, which are known as cross-feeding bacteria for butyrate production (42), were highly abundant only in enterotype B when treated with onion extracts (Fig. 3C).

**Fig 4:**
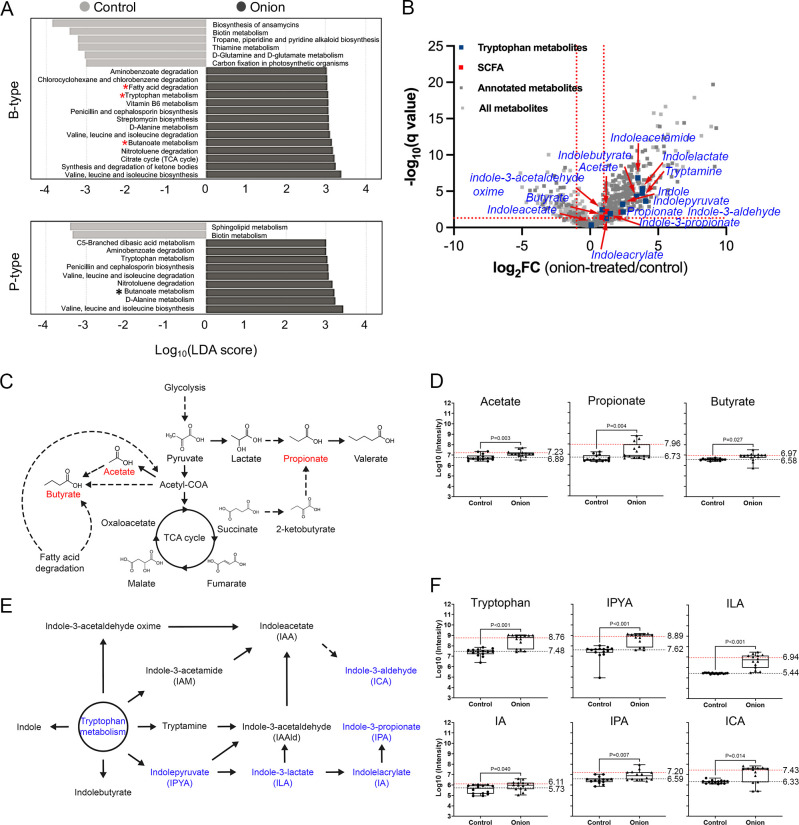
(**A**) Predicted KEGG functional pathway profiles that are distinctly present in each group. Bar chart represents differentially abundant KEGG pathways that were selected based on the following criteria: LDA score > 3 and adjusted *P* value of <0.05. The pathways that overlap with metabolites of interest in the following panels are indicated with red and black asterisks. (**B**) Volcano plots of differentially enriched metabolites between control and onion-treated groups. All metabolites and those with annotations based on publicly available database, such as KEGG and Human Metabolome Database, are indicated by light and dark gray circles, respectively. The metabolites that are overlapped with predictive functional KEGG pathways generated using 16S *rRNA* data are shown in red and blue circles. Statistical significance (adjusted *P* < 0.05) was determined based on unpaired *t*-test after two-stage step-up correction (indicated by horizontally dotted red line). (**C**) A schematic representation of the short-chain fatty acid (SCFA) biosynthesis pathways. Letters colored by red are metabolites that are highly enriched in the onion-treated group. (**D**) Comparison of amounts of SCFAs between the control and onion-treated groups. (**E**) A schematic representation of tryptophan metabolism and Stickland fermentation pathways (blue letters). (**F**) Comparison of amounts of tryptophan metabolites between the control and onion-treated groups. The red dotted line represents the mean of the onion-treated group, while the black dotted line represents the mean of the control group.

To predict KEGG functional bacterial gene profiles from the 16S *rRNA* data set, we used PICRUSt2. To determine whether distinct bacterial genes are enriched in response to onion extracts, we performed a pairwise comparison between the control group and the onion extract-treated group. The onion-treated group showed significant enrichments of genes related to branched amino acid metabolism (valine, leucine, and isoleucine), the tricarboxylic acid (TCA) cycle, antibiotic biosynthesis (penicillin, cephalosporin, and streptomycin), aminobenzoate/chlorocyclohexane and chlorobenzene/nitrotoluene degradation, tryptophan/vitamin B6/butanoate metabolism, and fatty acid degradation compared to the control group (Fig. 4A). Notably, the significant enrichment of pathways related to fatty acid degradation and butanoate metabolism corresponds with the taxa enriched in response to onion treatment (Fig. 3 and 4A).

### Metabolic profiles in response to onion extracts

To identify metabolites derived from gut microbiota in response to onion extracts and address the limitations of genomic analysis from 16S rRNA sequencing, we conducted an untargeted metabolomic analysis of fecal contents using LC-MS/MS. A total of 1,280 metabolites were detected after identification processing (Fig. 4B, light gray squares; Table S1). Among these, 425 metabolites were annotated based on the KEGG metabolism database or HMDB (Fig. 4B, dark gray squares; Table S1). Notably, levels of short-chain fatty acids, such as acetate, propionate, and butyrate, along with related metabolites like lactate, pyruvate, 2-ketobutyrate, malate, and fumarate, were significantly increased in response to onion treatment in the *Bacteroides* type (Fig. 4B, red squares and C). This increase is consistent with PICRUSt2 predictions for butanoate metabolism and fatty acid degradation (Fig. 4A).

In metabolic profiles, tryptophan-derived metabolites catabolized by gut microbiota, such as indole, tryptamine, and 3-methylindole, along with various indole derivatives, were highly overrepresented in the onion-treated group (Fig. 4B, blue squares). Notably, indole derivatives produced from Stickland fermentation, including indolepyruvate, indolelactate, indolepropionate, and indoleacrylate, were elevated in the onion-treated group compared to the control group (Fig. 4E and F). Importantly, IPA and ILA have been shown to possess anti-inflammatory properties that can mitigate intestinal inflammation (15).

We also profiled additional beneficial metabolites that increased in response to onion treatment. Polyphenols, such as caffeic acid, cinnamic acid, catechol, gallic acid, and alpha-curcumene, were highly enriched in the onion-treated group compared to the control (Table S1, beneficial metabolites list). These compounds are reported to play roles in neurodevelopment, alleviating depression (43), anti-diabetic activity (44), anti-cancer effects (45); promoting the growth of probiotics such as *Lactobacillus acidophilus* and *Lacticaseibacillus rhamnosus* (46); and preventing the progression of Parkinson’s disease (47, 48). Additionally, B-vitamins, including vitamins B3 (nicotinic acid), B5 (pantothenic acid), and B7 (biotin), were significantly enriched in the onion-treated groups (Table S1, beneficial metabolites list). These vitamins are known to alleviate severe colitis and diarrhea (49) and promote the growth of *Lactobacillus murinus*, which reduces mice intestinal ischemia/reperfusion injury by suppressing inflammation (50, 51).

### Enhancing probiotic growth with onion extract treatment

**Fig 5:**
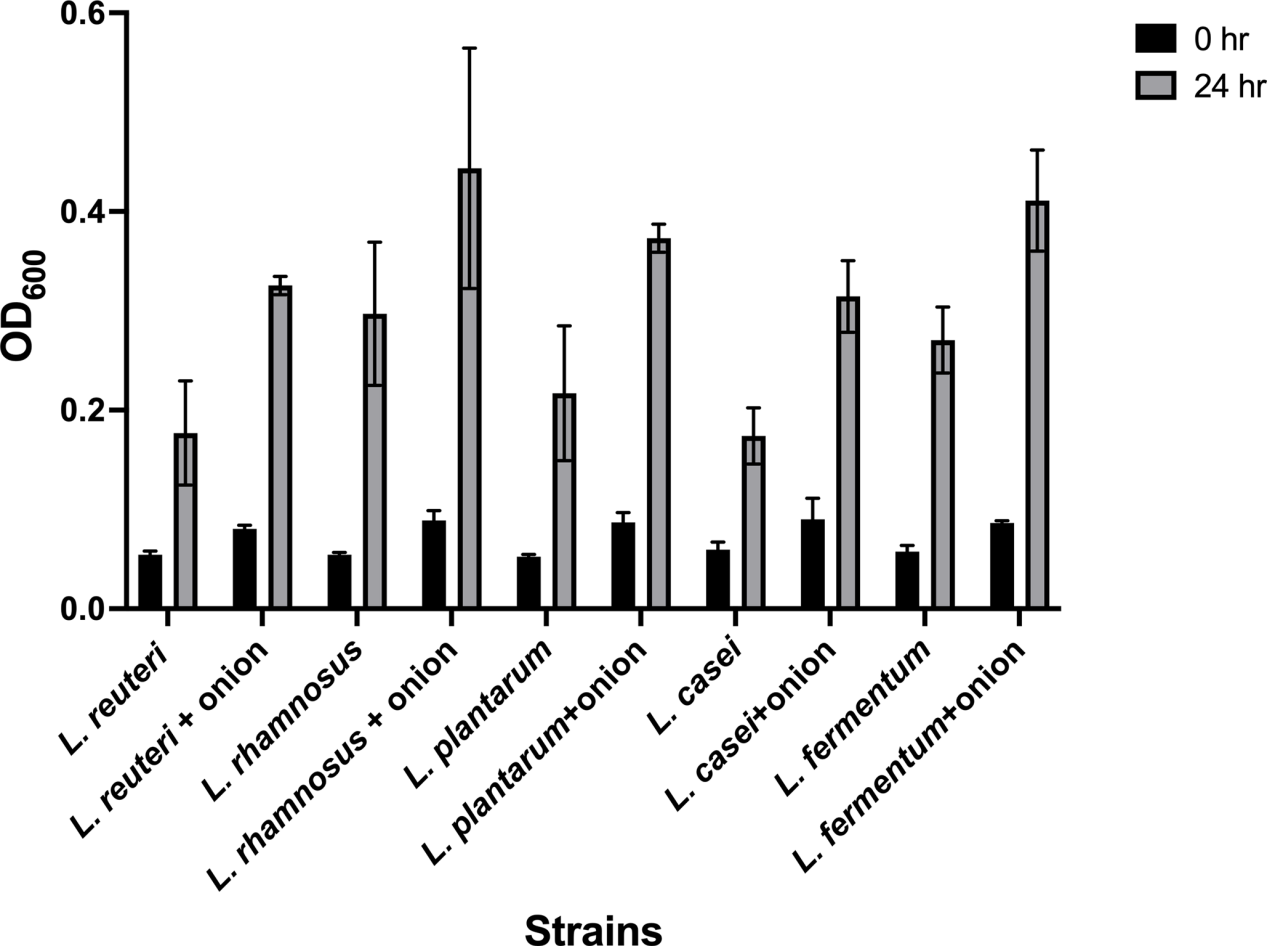
The growth enhancement of probiotics in the presence of onion extracts for 24 h. All strains were inoculated in a microplate and preactivated through incubation for 4 days. After growing for 4 days, the bacteria were serially diluted and incubated for 24 h in the presence or absence of onion extracts. The absorbance at 600 nm was measured at 0 or 24 h post-incubation. Data shown and error bars are mean with range; mean of two biological replicates.

Our results demonstrate the potential of onion extract as a prebiotic, significantly impacting gut microbial structure and metabolite profiles and promoting the production of beneficiary metabolites for human health (Fig. 2 to 4). To further investigate its ability to enhance probiotic growth, we conducted *in vitro* growth assays using *Lactobacillus* spp., which are recognized as beneficial probiotics for health. The species selected for this study included *Lactobacillus reuteri*, *L. rhamnosus*, *Lactobacillus plantarum*, *Lactobacillus casei*, and *Lactobacillus fermentum* (52–56). All tested strains, listed in Table 3, exhibited increased growth in the presence of onion extract compared to the control condition without the extract (Fig. 5). These findings suggest that onion extract holds promise as a potential prebiotic.

**TABLE 3:** *Lactobacillus* strains used in the experiment

Strain name^*a*^	Source or reference
*Lactiplantibacillus plantarum* KCTC 3108	(57)
*Lacticaseibacillus casei* KCTC 3260	(58)
*Limosilactobacillus reuteri* KCTC 3594	(55)
*Lacticaseibacillus rhamnosus* ATCC 53103 (LGG)	ATCC
*Limosilactobacillus fermentum* SJ_F2	This work

^a^ ATCC, American Type Culture Collection; KCTC, Korean Collection for Type Cultures.

## DISCUSSION

In this study, we employed gut enterotyping to reduce inter-individual variability, which often complicates data interpretation. By introducing enterotyping, we were able to effectively characterize the impact of onion extracts as a promising prebiotic. Specifically, onion extracts were shown to alter gut microbial structure (Fig. 2 and 3), leading to increased production of beneficial metabolites, such as butyrate, IPA, and ILA (Fig. 4). We reason that these changes are attributable to onion-derived prebiotic components, as detailed in Table 4, including FOSs and polyphenols, which are known to influence the gut microbiome (59–61). Additionally, onion extracts promoted the growth of lactobacilli prebiotics (Fig. 5), further supporting their role as effective prebiotics. In this study, we utilized enterotyping to stratify the population and assess the effects of onion-derived prebiotics. This stratification not only uncovers patterns that might be obscured by individual variability but also highlights the potential influence of dietary components on specific microbial compositions and their functional roles in gut health.

**TABLE 4:** Constituents and their composition of onion extracts used in this study

Composition	Onion (%)
Polysaccharides	42.5
FOS	12.6
Monosaccharides (Xyl, Ara, Glc, Fuc, and Suc)	18.8
Uronic acid	2.9
Polyphenols	1.2
Amino acids	12.0
Fatty acids (organic acids)	10.0
Total	100.0

Our results demonstrated that certain bacterial taxa, including *Bifidobacterium*_388775, *Feacalibacterium*, *Fusicatenibacter*, and *Lachnospiraceae*, were overrepresented exclusively in enterotype B and not in enterotype *P* (Fig. 3B). To explore the relationships between the observed certain taxa and enterotype B, we performed co-occurrence network analysis between *Bacteroides* and co-occurring genera. In our data set, *Bacteroides* showed a significant negative correlation with *Fusicatenibacter*, *Bifidobacterium*_388775, *Feacalibacterium*, and *Lachnospiraceae*. In contrast, no co-occurrence between these taxa enriched in enterotype B and *Prevotella* was observed after onion treatment (Fig. S3). However, the small number of subjects in enterotype *P* (*n* = 5) limits the robustness of our co-occurrence analysis, and a larger sample size is needed for more conclusive results.

In response to onion extract treatment, specific taxa such as *B. adolescentis* and *F. prausnitzii*, known for their butyrogenic capabilities, were significantly enriched (Fig. 3C). These bacteria rely on cross-feeding mechanisms to produce butyrate, a key metabolite linked to gut health (42). This enrichment aligns with the activation of metabolic pathways like fatty acid degradation and butanoate metabolism, which were significantly upregulated in the onion-treated group, suggesting a functional impact of onion-derived prebiotics, including FOS and polyphenols, on the gut microbiota (Fig. 4A). In line with the observations from 16S rRNA analysis, metabolite analysis further confirmed these results, showing significantly elevated levels of butyrate in response to onion treatment (Fig. 4C and D). Previous studies have demonstrated that butyrate is a crucial molecule to regulate energy metabolism for gut homeostasis. The underlying mechanism by which butyrate preserves gut homeostasis involves its transport to mitochondria, where it scavenges oxygen through b-oxidation, helping to control oxygen level in the gut (62). This finely tuned oxygen level can prevent opportunistic pathogens, such as *Salmonella* Typhimurium, from infecting the gut by depriving them of the oxygen needed for aerobic respiration (63). Furthermore, it has been noted that butyrate plays a role in downregulating inflammation by inducing the differentiation of regulatory T cells in the intestine (64).

It should be noted that our results showed a significant increase in the production of IPA and ILA in response to onion extract treatment (Fig. 4). Recent studies have emphasized the importance of tryptophan metabolism-derived indole derivatives, such as IPA and ILA. These compounds play crucial roles in various host biological processes, including the maintenance of epithelial barrier integrity, modulation of immune responses, protection against pathogens, and the regulation of inflammation and metabolic disorders (17–20). Previous findings have also shed light on the inter-microbial crosstalk involved in the production of microbiota-derived indole derivatives through probiotic supplementation. Notably, the increased production of ILA, a precursor to IPA, can be enhanced by the addition of a probiotic *L. reuteri*, which upregulates mRNA expression of acyl-CoA dehydrogenase (ACD) and indolelactate dehydrogenease, thereby facilitating IPA production (15). In our study, we observed an enrichment of *Bifidobacterium* and *Faecalibacterium* in the onion-treated group (Fig. 3). Given that *Bifidobacterium* is known as an ILA producer (65) and *Faecalibacterium* encodes the ACD gene (15), the observed increase in IPA production can be plausibly explained by the presence of these bacteria in the onion-treated group.

Recent study has unveiled the role of inter-microbial crosstalk in the production of microbiota-derived indole derivatives by prebiotic supplementation. In defined gut-derived bacterial community comprising *Bacteroides thetaiotaomicron*, *Escherichia coli*, and *Clostridium sporogenes*, the supplementation of a prebiotic, such as dietary fiber, has been shown to induce the production of ILA and IPA (16). The underlying mechanism involves the decomposition of dietary pectin by *B. thetaiotaomicron*, leading to the production of monosaccharides such as arabinose, rhamnose, xylose, and galacturonic acid. These sugars repress the transcription of *tnaA* (tryptophanase) of *E. coli* likely through catabolite expression, thereby increasing the availability of tryptophan to Stickland fermenter *C. sporogenes* in the gut environment. However, in more complex gut microbiota, IPA was not detected across all samples, and no known IPA producers or *Bacteroides* spp. were identified in the communities (16), highlighting the challenges in extrapolating *in vitro* and animal study results to the human context. Consistent with this, our data set showed no change in the abundance of *Bacteroides* and no detection of known IPA producers in the onion-treated group compared to the control group (Fig. 3). Interestingly, we reason that the sporadic detection of IPA in human gut microbiota could be attributed to inter-individual variability because IPA was detected across all participants in our study (Fig. 4F), suggesting that such variability might explain the inconsistent presence of IPA in human samples. This observation further underscores the importance of gut enterotyping as a strategy to reduce variability and obtain more consistent results. While the smaller sample size of the *Prevotella*-dominant group in this study represents a limitation, further studies with larger *Prevotella*-dominant cohorts are necessary to strengthen the robustness of enterotype-specific conclusions.

It should be noted that the present study has a limitation regarding bioaccessiblity of onion powder. A previous study demonstrated that an *in vitro* fecal incubation model can maintain viability, diversity, and compositional and functional profiles of the inoculum microbiome, though it has limited detection capacity for low-abundance species (31). Given that food samples undergo the entire digestive tracts—passing through oral, gastric, and intestinal digestion (66)—the effects of onion powder on the gut microbiome should be assessed after simulating this digestive pathway, as the powder would likely not remain intact following digestion.

In conclusion, our observations suggest that onion extract has significant potential as a prebiotic. It contains various beneficial constituents, including well-known prebiotic FOS, as detailed in Table 4. Furthermore, onion extract significantly influences the structural changes in gut microbiota, promotes the production of beneficial post-biotics, and enhances the growth of probiotics. These findings indicate that onion extract could be an effective dietary component for supporting gut health.

## Data Availability

A Strengthening the Organizing and Reporting of Microbiome Studies checklist (version 1.03) is available at dx.doi.org/10.6084 /m9.figshare.26928916. All amplicon sequence data and metadata have been made public through the EMP data portal (Qiita, https://qiita.ucsd.edu; study ID: 15658) accessed on 20 August 2024.

## SUPPLEMENTAL MATERIAL

Supplemental figures msystems.01189-24-s0001.docx Figures S1-S3.

Table S1 msystems.01189-24-s0002.xlsx Metabolite profiles based on LC-MS/MS analysis.

ASM does not own the copyrights to Supplemental Material that may be linked to, or accessed through, an article. The authors have granted ASM a non-exclusive, world-wide license to publish the Supplemental Material files. Please contact the corresponding author directly for reuse.
