# Supplementary material for: The prebiotic potential of dietary onion extracts: shaping gut microbial structures and promoting beneficial metabolites
Source: mSystems. 2024 Dec 23;10(1):e01189-24. doi: 10.1128/msystems.01189-24 (PMC11748487; doi:10.1128/msystems.01189-24)
Supplement: Supplemental figures — Figures S1-S3. [file msystems.01189-24-s0001.docx]

**Supplementary materials**

**The Prebiotic Potential of Dietary Onion Extracts: Shaping Gut Microbial Structures and Promoting Beneficial Metabolites**

**Running title: Prebiotic Effects of Onion Extracts**

Yebeen Yoo^1†^, Seongok Kim^1,2†^, WonJune Lee^2^, Jinwoo Kim^1,2^, Bokyung Son^3^, Kwang Jun Lee^4*^, Hakdong Shin^1,2*^

^1^ Department of Food Science & Biotechnology, College of Life Science, Sejong University, Seoul 05006, Republic of Korea

^2^ Carbohydrate Bioproduct Research Center, Sejong University, Seoul 05006, Republic of Korea

^3^ Department of Food Biotechnology, Dong-A University, Busan 49315, Republic of Korea

^4^ Center for Infectious Diseases Research, Division of Zoonotic and Vector Borne Diseases Research, National Institute of Health, Cheongju 28159, Republic of Korea

^†^These authors contributed equally to this work

* Correspondence:
Kwang Jun Lee, PhD (kwangjun@korea.kr)

Hakdong Shin, PhD (hshin@sejong.ac.kr)


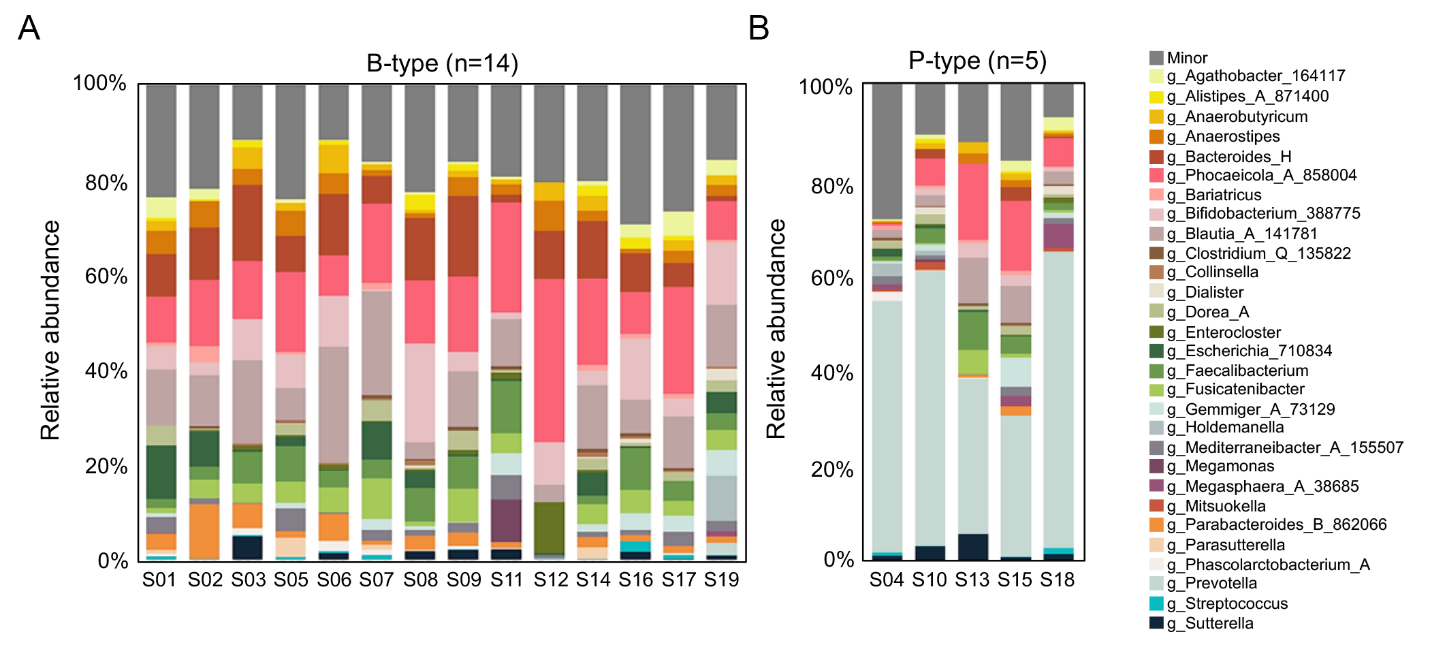
Figure S1. Changes in individual microbial composition of baseline samples at the genus level across all participants in each enterotype.


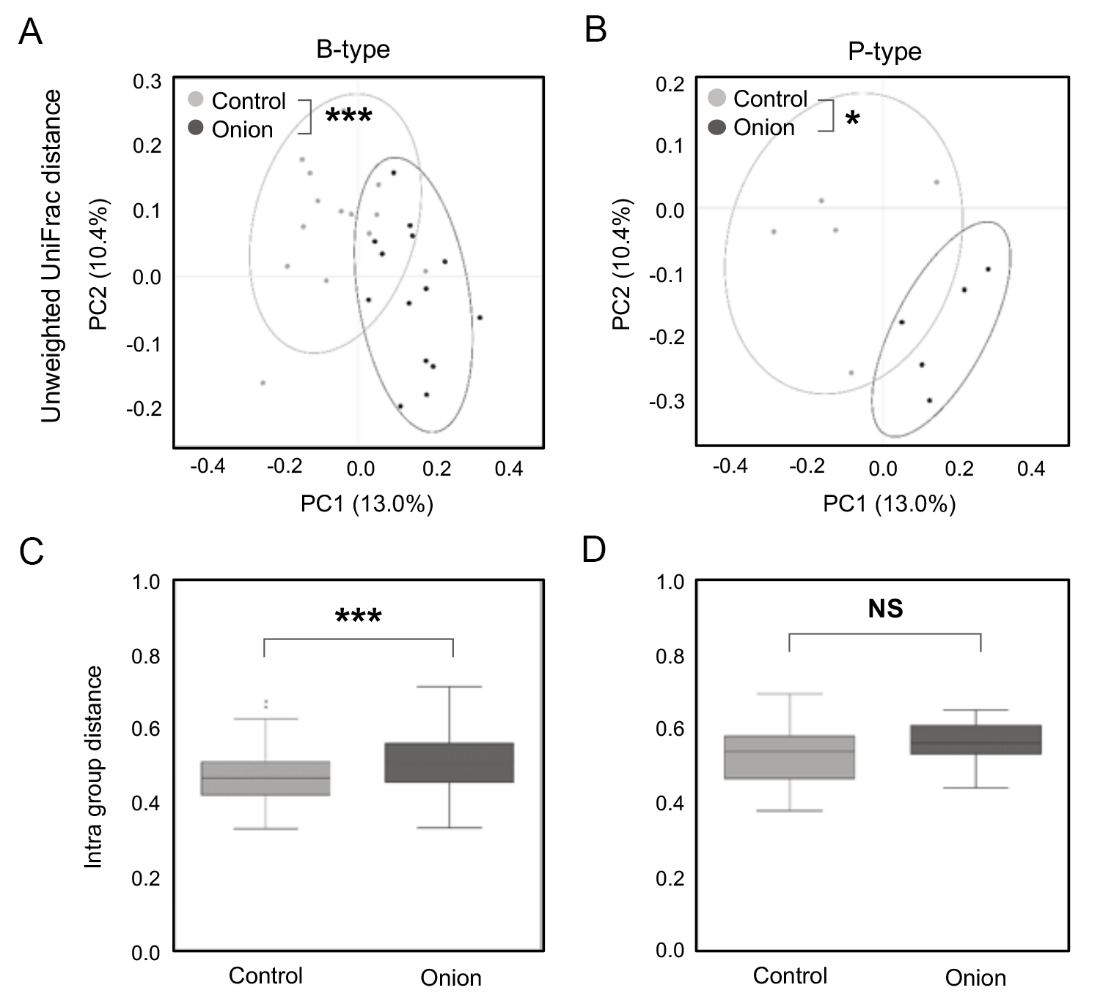


Figure S2. The effect of onion extracts on changes in gut microbial structure according to enterotype. (A and B) Beta diversity evaluated by weighted UniFrac distance in B type (A) and P type (B), respectively. PERMANOVA was used to test statistical differences between control and onion-treated groups; Mann-Whitney test; *p<0.05 and ***p<0.001. (C and D) Intragroup distance using weighted UniFrac distance between groups in B type (C) and P type (D), respectively. Data shown and error bars are mean ± SEM (non-parametric t-test, ***p<0.001).


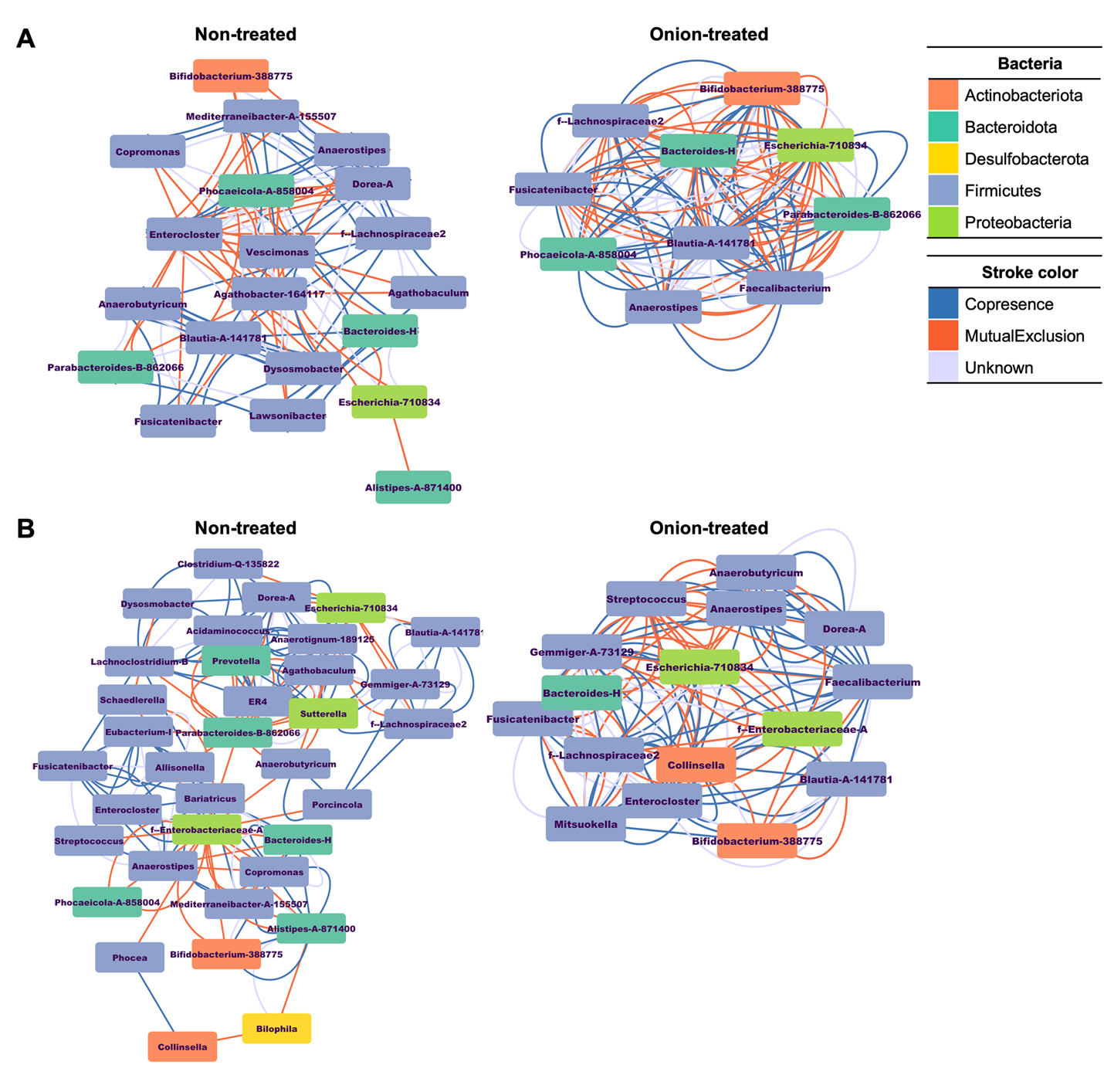


Figure S3. Co-occurrence networks of each enterotype between control and onion-treated group. The co-occurrence networks show correlations of bacterial taxa in either enterotype B (A) or enterotype P (B) non-treated (left) and onion-treated (right). Nodes are colored according to their corresponding phylum, with blue edges indicating positive correlations (co-presence) and red edges indicating negative correlations (mutual exclusion)
